# Covert pre-leukaemic clones in healthy co-twins of patients with childhood acute lymphoblastic leukaemia

**DOI:** 10.1038/s41375-022-01756-1

**Published:** 2022-12-20

**Authors:** Anthony M. Ford, Susan Colman, Mel Greaves

**Affiliations:** Centre for Evolution and Cancer, The Institute of Cancer Research, London, UK

**Keywords:** Acute lymphocytic leukaemia, Cancer genetics

Approximately 1% of births involve twins. Around half of these are identical or monozygotic and a similar fraction will be represented in children with leukaemia. Despite this rarity Acute lymphoblastic leukaemia (ALL) in patients who are monozygotic, or identical, twins has provided an ‘experiment of nature’ providing remarkable insights into the origins and natural history of the most common cancer in children [1, 2]. The high concordance for ALL in identical twins, not seen for other paediatric cancers, has long been recognised and its biological basis identified by genomic studies on the leukaemic cells of twin pairs.

The sharing of identical but non constitutive (germ line), clone specific markers including leukaemia fusion gene genomic sequences or *IGH* rearrangements in the leukaemic cells of twin pairs with ALL [1, 2] provided compelling evidence for a common, clonal (single cell) origin in one twin in utero. This supported the much earlier speculation [3, 4] that concordance might reflect the blood cell chimaerism that follows as a consequence of intraplacental anastomoses. This interpretation is further endorsed by the observation that cases of concordant ALL occur only in monozygotic twins that are also monochorionic or with a single placenta, except in very rare pairs with dichorionic but fused placentas where blood exchange is possible [1]. Only some 60% of identical twins have a single placenta, this variable feature being dependent on timing of splitting of the single early zygote [5].

Other ‘backtracking’ studies on the genomics of childhood ALL have shown that the same clonal markers exploited in twin cases are present in the blood at birth in the majority of singletons with ALL establishing that this cancer is commonly initiated in utero [2, 6]. Most paediatric cancers probably share a similar in utero origin in the developing embryo or foetus. The restriction of high concordance rates in twins to leukaemia likely reflects that this cancer type disseminates covertly into the blood circulation at an early ‘pre-leukaemic’ stage of its pre-natal clonal evolution, and up to 14 yrs, [7–9] before progression leads to a post-natal diagnosis of overt ALL.

Uncertainties arise however from the actual concordance rates. Infants (<18months) have a distinctive subtype of ALL with a pro-B immunophenotype and *KMT2A* fusion genes and the concordance of this subtype in infant twins that are both monozygotic and monochorionic is very high and likely close to 100% [1]. This high rate is compatible with the pre-natal origins of *KMT2::AFF1* fusion in infant ALL [10, 11] and suggests that leukaemogenesis in this unique subtype has a rapid, ‘big bang’ evolutionary trajectory and is complete or irreversible by the time of birth. This might then reflect that a single chromosomal aberration, *KMT2A* gene fusion, may, unusually, be sufficient for malignant cancer [2, 12]. The genomics of infant ALL with *KMT2A* fusions is compatible with this notion. Additional genetic abnormalities do occur, but these are sub clonal (<30% allele burden) and often absent in relapse [13, 14].

In contrast, for older (2–15 yr) twin children with the more common subtypes of B cell precursor ALL, the concordance rate has been calculated to be significantly lower at around 15% before correction for placental status [1]. These age-associated differences in concordance prompted the suggestion that whilst infant ALL might be pre-natal in origin the disease could be mostly post-natal in older children [4]. Or, in other words, the ~85% that are discordant might be because these cases are initiated post-natally.

An alternative explanation for discordance in older twin children is more in keeping with current understanding of the genomics and clonal evolution of the common subtype of B cell precursor ALL in children [2]. This indicates a requirement for one or more additional mutations to complement the initiating lesions of *ETV6::RUNX1* fusion or high hyperdiploidy and which commonly arise post-natally within a persistent but clinically covert pre-leukaemic clone spawned pre-natally. These recurrent, secondary genetic events are mostly RAG driven copy number changes (deletions) in the case of *ETV6::RUNX1* + ALL [15, 16] and high hyperdiploid ALL [17] plus RTK-RAS mutations in high hyperdiploid ALL [17]. In identical twins that are concordant for ALL the secondary genetic lesions are distinct and individual for each twin reflecting independent post natal clonal evolution [18, 19].

This then predicts that discordant cases of childhood ALL also originate *in utero*, but discordance is for the ‘second hit’, which occurs in one twin only. If correct, healthy co-twins of patients with ALL should harbour silent pre-leukaemic cells with the same initiating lesions and other clonal markers (IGH) as in the co-twin with clinical ALL but lacking the secondary genetic lesions detected in the twin with ALL.

Distinguishing between these alternative explanations for clinical discordance of ALL in twins is of some importance for our understanding of the natural history of ALL as well as for clinical management, risk assessment and counselling to parents of twins.

## Patients

We have previously published some anecdotal evidence on individual twin pairs, which indicated that the healthy co-twin of a patient with ALL can have putative pre-leukaemic cells in their blood (see Table 1 and references therein). We now report a more systematic examination of this question in those twin pairs plus an additional three pairs providing a unique set of 8 monozygotic twin pairs with discordant B cell lineage ALL (Table 1). In all cases informed consent from parents was obtained and genetic screening was covered by our local clinical research ethics committee approval. The twin pairs have been followed up for varying amounts of time and up to 15 years (pair 1 in Table 1), in some cases with serial testing in concert with their regular clinical and haematological assessment (pairs 1, 3, 4 and 5 in Table 1). To date, two twins that were clinically healthy at the time of their twin sibling’s diagnosis of ALL have themselves developed ALL (pairs 6 and 7 in Table 1). Identical twin children can also be concordant for AML [20] and the pre-natal origins of at least some childhood AML was confirmed by neonatal blood spot screening for *RUNX1::RUNX1T1* (*AML1*::*ETO*) [21]. We include in Table 1 one monozygotic, monochorionic twin pair (8 A/B) who were discordant for AML.

**Table 1:** Characteristics of identical twin pairs.

Twin Pair	Sex	Genetics	Twin A (age at dx)	Twin B (age at dx)	Pre-leukaemic cells in healthy co-twin (B) twin detected by:	Age of healthy co-twin at last follow up/screening*	Reference
1 A/B	F	*ETV6::RUNX1*	2 y	N/A	FISH, *IGH* (*DJ*), Q-PCR, time course and Guthrie	14 y 7 m	23, 24, present study
2 A/B	F	*ETV6::RUNX1*	3 y 9 m	N/A	FISH and Guthrie	4 y	present study
3 A/B	F	*BCR::ABL1*	5 y	N/A	FISH, Q-PCR, time course and Guthrie	9 y 8 m	18
4 A/B	F	*RANBP2::ABL1*	1 y 1 m	N/A	Q-PCR, time course and Guthrie	3 y 5 m (ongoing)	present study
5 A/B	M	High Hyperdiploid	5 y	N/A	FISH, *IGH* (*DJ*), Q-PCR	6 y 11 m	26
6 A/B	F	*KMT2A::AFF1*	9 m	10 m	Southern blot	10 m	11
7 A/B	F	*ETV6::RUNX1*	5 y 2 m	13 y 11 m	PCR & sequencing	5 y 2 m**	8
8 A/B***	M	*KMT2A::MLLT10*	3 y	N/A	PCR & sequencing	3 y 3 m	7

*Decision not to proceed with regular re-screening or to stop screening (pairs 1, 3 & 5) was taken by parents in consultation with their child’s clinician.

**Retrospective testing of sample taken from twin 7B at diagnosis of sibling: 5 yr 2 m.

***Case 8 A was AML. All other cases were B lineage ALL.

## Screening

**Fig. 1:**
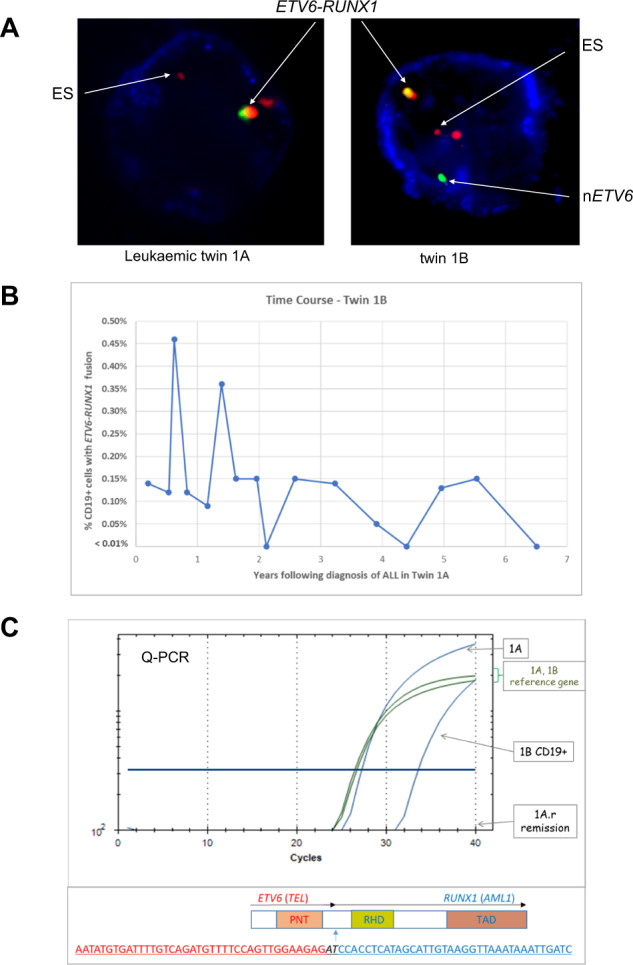
Pre-leukaemic cells in blood of the healthy co-twin of a patient with t(12;21) ALL (pair 1A/B). **A** MI-FISH (Multi- (3) colour Immuno-FISH), for *ETV6::RUNX1* translocation and CD19 protein in PB mononuclear cells from twins 1A (leukaemic) and 1B respectively. CD19 is indicated by blue staining, *ETV6* by green, *RUNX1* by red and *ETV6::RUNX1* fusion by the green-red signal. ES marks the LSI *ETV6::RUNX1* extra signal from the Dual Colour Translocation Probe (Vysis, Abbott Molecular). In all discordant PB screens, B-lineage cells were enriched with a CD19 immunomagnetic sort (Miltenyi Biotec) before staining. In each case we scrutinised at least 1000 CD19 + cells per time point. Cells scoring positive for the fusion signal (or high hyperdiploidy) were very infrequent: between 1 and 5 per sample. There we no false positives in control samples with the probes used and the scoring criteria applied ie a two colour fusion signal plus extra residual signal from the translocated *RUNX1*. **B** Time course of levels of the *ETV6::RUNX1* fusion gene FISH positive cells observed in PB of twin 1B. **C** Upper Panel: Patient-specific fusion gene Q-PCR profile of PB DNA isolated from the diagnostic twin (1A) and from CD19-enriched PB isolated from the sibling child (1B). No fusion signal was observed in the remission sample of the leukaemic twin (1A.r) after treatment. Lower Panel: shows the patient-specific *ETV6::RUNX1* genomic fusion sequence.

Parents were first asked about placental status at birth, checking obstetric records if necessary. All those twin pairs reported here had a shared or monochorionic (single) placenta. Cytogenetic data on the ALL-case sibling was accessed to identify candidate initiating lesions – gene fusions or high hyperdiploidy. These, along with *IGH* rearrangements provide, at the genomic sequence level, clone specific markers. If the pre-leukaemic cells are shared pre-natally, via intra-placenta exchange, then these markers should be present in the healthy co-twin’s blood and detectable at a level within the range of the screening methods. Screening of peripheral blood (PB), neonatal blood spots (Guthrie cards) or, in a single case, bone marrow (twins 7 A/B). was performed using different PCR methods and/or multicolour immuno-FISH (MI-FISH) on CD19 enriched cells as previously reported [6, 22, 23]. For putative pre-leukaemic cells to be scored as positive for *ETV6::RUNX1* by MI-FISH they must have a two-colour (red/green, appearing yellow) fusion signal plus a small red signal as remnant of the translocated *RUNX1* allele, with retention of one red and one green signal indicating the presence of the normal (untranslocated) *RUNX1* and *ETV6* alleles respectively (see Fig. 1A).

### Twin pairs

#### Pair 1A/B

We have previously reported a twin pair discordant for *ETV6::RUNX1* + ALL (Table 1, pair 1A/B). CD34 + /CD38 low/CD19 + cells (putative stem/B progenitor) from the healthy co-twin expressed *ETV6::RUNX1* mRNA and shared the same D-JH genomic *IGH* sequence as in the leukaemic cells of her sibling [23]. Using MI-FISH, we showed that the initiating *ETV6::RUNX1* gene fusion was present in the PB of both twins but only cells from the twin with ALL had the additional multiple “drivers” of leukaemia - including deletion of the normal copy of *ETV6*, deletion of *PAX5* and 10p gain [24].

We have monitored the levels of the putative pre-leukaemic cells in the blood of the healthy co-twin by MI-FISH over a period of 6.5 years and observe the continuous, albeit fluctuating, presence of low levels of fusion gene positive CD19 + cells (Fig. 1A, B).

We have also subsequently cloned the patient clone-specific *ETV6::RUNX1* genomic fusion from the overt ALL cells in twin 1A and, by Q-PCR and sequencing, confirm that the same or identical sequence is present in CD19 + cells of the healthy co-twin 1B (Fig. 1C) and in the neonatal blood spots of both twins.

#### Pair 3A/B

**Fig. 2:**
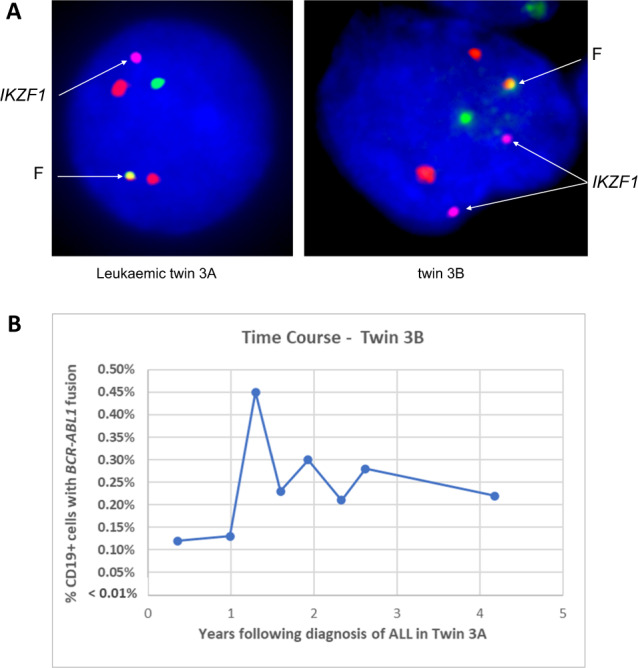
Pre-leukaemic cells in blood of the healthy co-twin of a patient with t(9;22) ALL (pair 3A/B). **A** FISH for *BCR::ABL1* translocation in PB mononuclear cells from twin 3A (leukaemic) and (CD19 + cells) from twin 3B respectively. *BCR* is shown by the green signal, *ABL1* by red, and *BCR::ABL1* fusion (F) by the green-red signal (Vysis, Abbott Molecular). Blue staining is DAPI. Two copies of the *IKZF1* gene (http://bacpac.chori.org, purple signal) are present in twin 3B but only one copy in the diagnostic child. **B** Time course of levels of the *BCR::ABL1* fusion gene FISH positive cells in PB of twin 3B.

We performed a similar MI-FISH time-course analysis for *BCR::ABL1* fusion+ cells in monozygotic twins (pair 3A/B) discordant for *BCR::ABL1* ALL [18]. The healthy co-twin in this pair was previously found to have detectable clone-specific *BCR::ABL1* genomic sequence in her neonatal blood spot and *BCR::ABL1* MI-FISH positive cells in a single blood sample screened 4 months after her sibling was diagnosed with ALL [18]. Levels of the fusion gene in the PB of the healthy co-twin were again observed to fluctuate for over 4 years post diagnosis of the leukaemic twin and to exist for at least 9 years after birth (Fig. 2A, B), without transformation to overt leukaemia.

#### Pair 4A/B

**Fig. 3:**
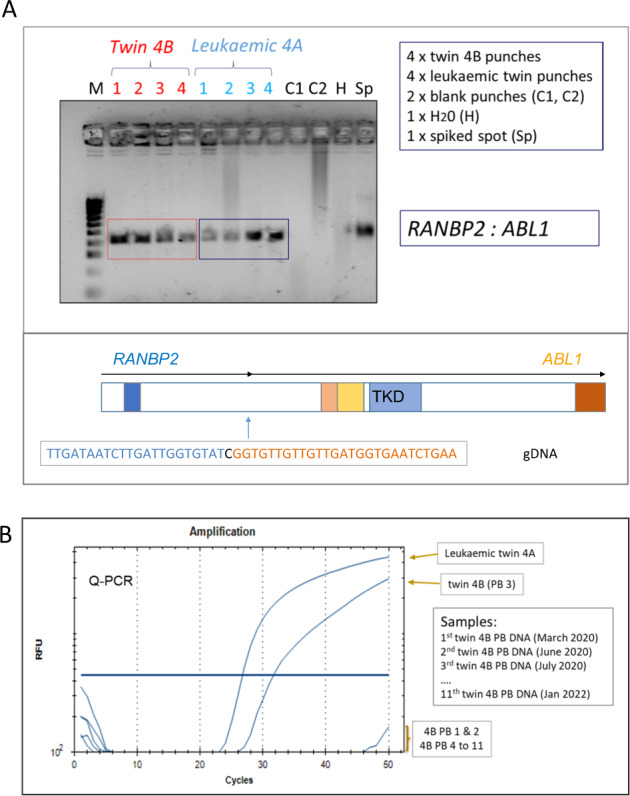
Pre-leukaemic cells in blood of the healthy co-twin of a patient with t(2;9) ALL (pair 4A/B). **A** Upper Panel: Patient-specific fusion gene profile of DNA isolated from neonatal blood spot punches taken from the Guthrie cards of twin 4A (leukaemic) and 4B respectively. Lower Panel: the patient-specific *RANBP2::ABL1* genomic fusion sequence. **B** Q-PCR time course of levels of the *RANBP2::ABL1* fusion gene observed in serial PB sampling of twin 4B. The patient-specific fusion gene was used as the probe. Only one positive sample was observed (PB 3).

A third pair investigated are previously unreported (Table 1, pair 4A/B). These were an infant twin pair discordant for t(2;9)/*RANBP2::ABL1* ALL. The rare, high-risk fusion joins the *RANBP2* and *ABL1* genes leading to the activation of the tyrosine kinase domain of ABL1 [25]. We first confirmed the *RANBP2::ABL1* fusion at diagnosis by cDNA analysis with primers spanning between exon 16 of *RANBP2* and exon 2 of *ABL1* and then cloned the patient-specific genomic fusion. The specific fusion sequence was detectable in the neonatal blood spots of both the sibling with ALL and her healthy co-twin (Fig. 3A). We have screened the blood cells of the healthy co-twin three monthly for two years (to date) for the genomic sequence by Q-PCR. Only one sample, the third, registered positive (Fig. 3B).

### Data on other twin pairs

#### Pair 2A/B

Twin 2A had *ETV6::RUNX1* + ALL (unreported). In the single sample of PB accessed from the healthy co-twin we detected a similarly low level of fusion gene positive cells (0.02%) as in other cases. The product of the reciprocal der(12)t(12;21) genomic, *RUNX1::ETV6*, was cloned from the diagnostic DNA (twin 2 A) and the identical fusion sequence was identified in the Guthrie cards of both children.

#### Pair 5A/B

We previously reported this male discordant pair [26]. The healthy co-twin had cells that showed gain of X and triploidy for chromosome 18, as observed in his sibling’s ALL cells. In contrast to the latter, putative pre-leukaemic cells in the healthy co-twin lacked monoallelic deletion of *TCF3*, a presumed secondary (and post-natal) genetic lesion. Blood from the healthy co-twin was screened by MI-FISH four times over a 27-month period with cells in the range 0.12 to 0.27% having the chromosomal markers (+18 and +X) of the putative pre-leukaemic clone.

#### Pair 6A/B

This was the first twin pair we reported [11] and were infant girls (aged 9 months). One twin was diagnosed with t(4;11) and *KMT2A::AFF1* fusion (*MLL*::*AF4*) and the co-twin was clinically well with normal blood haematology. In view of the anticipated risk to the second twin a bone marrow sample was taken one month after the sibling’s diagnosis of ALL and found to contain elevated blast cells. We showed the same sample to have *KMT2A::AFF1* by Southern blot analysis. Repeating the Southern blot with three different restriction enzymes revealed the same sized fragments in both twins suggesting their clonal identity [11]. Based on this combined haematological and molecular evidence, and despite the lack of clinical symptoms or clear blood involvement, a diagnosis of ALL was made and treatment initiated. Both twins were treated with the same combination chemotherapy protocol (Chilean ALL 87, based on BFM 86) but their clinical outcome was different. The patient diagnosed first with clinical symptoms had two relapses and died. The sibling who was clinically discordant but then found to have the leukaemic clone had a sustained remission for 4 years [27] and remains disease free some 30 years after diagnosis (M-E Cabrera, personal communication). This case and two similar twin pairs reported [18] with different clinical outcomes suggests that early or pre-clinical detection of ALL cells might improve prospects for survival, especially perhaps in twin pairs clinically discordant for a high-risk subtype of ALL.

#### Pair 7A/B

This was an unusual but very informative case where screening was retrospective [8]. Twin A was diagnosed age 5 with *ETV6::RUNX1* + ALL. Her sibling, twin B, was diagnosed also with *ETV6::RUNX1* fusion some 9 years later (at age 14) and we obtained samples at that time point and cloned the *ETV6::RUNX1* genomic fusion sequence from twin 7B’s ALL cells. When that patient’s twin sibling (7 A) was diagnosed 9 years earlier a bone marrow aspirate and smear had been taken from her clinically well co-twin (7B) and considered haematologically normal. That smear had been retained and we were able to extract DNA which was found to contain the identical *ETV6::RUNX1* fusion sequence as in the twin’s diagnostic sample 9 years later [8]. Although the presence of putative pre-leukaemic cells was detected retrospectively, this twin pair indicate that albeit rarely, a second twin can develop ALL after a protracted latency. We cannot know whether the blood of that 5-year-old healthy twin would have contained detectable levels of pre-leukaemic cells as found in the bone marrow.

#### Pair 8A/B

This final monozygotic, monochorionic twin pair in the series were discordant for AML with *KMT2A::MLLT10* fusion [7]. Shortly after the diagnosis of AML in twin 8A, the healthy co-twin (8B) PB and neonatal blood spot were screened for *KMT2A::AFF1* fusion and found to be negative. The twins were 3 years old at the time of diagnosis of AML in twin 8A and we drew the tentative conclusion that, in contrast to infant cALL with *KMT2A* fusion this case might well have been postnatal in origin with no neonatal sharing of the pre-leukaemic clone. Encouraged by this possibility twin 8B served as a donor for a successful bone marrow transplant for the sibling with AML.

## Discussion

These data show that in cases of clinically discordant B cell precursor ALL in identical twins, the healthy co-twin has the identical clonotypic marker (fusion gene genomic sequence) of putative pre-leukaemic cells in their neonatal blood spots as in their sibling twin with ALL (4/4 cases tested positive). In all 6 twin pairs with clinically discordant ALL the healthy co-twin tested after the diagnosis of ALL in the twin sibling had low levels of blood cells (−0.1 to 0.4 % of CD19 + cells) with genetic markers indicative of the common clonal origin with the overt ALL in his/her twin sibling. This then confirms that, in common with concordant cases of childhood ALL in twins [1], the more frequent cases of clinically discordant ALL in monozygotic twins usually originate *in utero*. Restriction of a diagnosis of ALL to one twin most likely then reflects that the healthy co-twin’s silent pre-leukaemic cells fail to acquire the essential secondary genetic events for progression to overt ALL [2]. The low frequency of pre-leukaemic cells in healthy co-twins and lack of markers for physical sorting precludes genomic screening for driver mutations other than the shared initiating mutation, but they do lack the secondary mutations present in the overt leukaemic cells of their co-twin. This interpretation is also in line with the observation that covert pre-leukaemic clones are generated during normal foetal life a rate that far exceeds (~100 times) the frequency of clinical ALL [22, 28]. The higher, though still incomplete, penetrance of ALL in twin pairs of around 15% (but maybe ~25% for monochorionic cases) may reflect that identical twins share the same genetic background along with other social or lifestyle factors that contribute to risk of ALL [2].

In the healthy co-twins of patients in which serial assessment of blood pre-leukaemic cells (4 cases) was possible it appears that this population is relatively stable or persistent, albeit at a low and fluctuating level in blood. This implies continued risk of ALL for that child. In most cases where ALL is concordant, the two diagnoses are within 12 to 18 months of each other [1] but the occasional much later second diagnosis - 9 years in one twin pair (pair 7 A/B, [8]), testifies to the persistence of a functional pre-leukaemic clone.

Our series of cases of discordant ALL in identical twins is perhaps unique but a few other individual pairs have been recorded in which the healthy co-twin was evaluated for the presence of clinically silent pre-leukaemic cells. Chuk et al. [29] described a pair of monozygotic infants that were discordant for ALL with *KMT2A::MLLT1* + (*MLL::ENL*) ALL, diagnosed in one twin aged 9 months. The *KMT2A::MLLT1* fusion was also present in the blood and bone marrow of the healthy co-twin at 7 and 10 weeks respectively after diagnosis of ALL in the sibling, but was no longer detectable in blood, by RT-PCR, by 14 to 20 weeks. Disappearance of the pre-leukaemic signal in the healthy co-twin’s blood was preceded by a viral neutropenia which the authors speculate could have triggered immune removal of pre-leukaemic cells. The co-twin remained healthy and ALL free at three years of age indicating that concordance of infant ALL with a pre-natal *KMT2A* fusion though very high, is not universal.

In another discordant twin pair, one twin was diagnosed with *KMT2A::AFF1* + pro-B ALL age 5 months [30]. The fusion gene was however absent from the neonatal blood spots of both twins, but they did share an identical *NRAS* mutation suggesting this, rather than the *KMT2A* fusion, may have been the pre-natal initiating event.

These observations raise some practical issues that follow when a twin is diagnosed with leukaemia. Parents have the additional burden of concern that the other twin will be at risk and need informed advice. We are aware of parents being told the risk to the second twin (in a typical case of B cell precursor ALL) was one in a million, and in another case (of T-ALL), inevitable or 100%; both very wide of the mark. We now advise first ascertaining that the twins are identical, with genetic screening if necessary. Non identical twins will have the sibling risk which is only slightly elevated over the average (around three-fold from a base rate of ~1 in 2000) [31]. The next step should be to determine placental status at birth, through parental recall and/or obstetric records. Except in the rare instance of two fused placentas, the elevated risk to the second twin is minimal in dichorionic, identical twin pairs. Where the twins are both monozygotic and monochorionic, the substantially increased risk comes into play.

Risk level for concordance depends on subtype of leukaemia. For infants (<18months) with pro-B ALL and *KMT2A* fusion that risk level is very high, and the healthy co-twin should have immediate blood tests with a follow up bone marrow.

For twin patients where one is diagnosed with the more common subtype of B cell precursor ALL, the risk level conveyed to parents should be in the range of 15–25%. If the co-twin is clinically well there is a case for clinicians and parents to consider for examination of blood for clonal markers of leukaemia cells that were present in the twin diagnosed with ALL. Markers chosen should be those likely to be early or initiating mutations (eg, fusion genes or high hyperdiploidy). Alternatively, *IGH* and inappropriate *TRD* rearrangements can be used as clone-specific markers. But here some caution is necessary as the rearrangement process continues to diversify in leukaemic clones. An analysis of *IGH* rearrangement patterns in a set of twins with concordant ALL revealed that both twins’ leukaemic cells retained an IGH marker of the initially transformed single cell which is a unique D-JH sequence at the *IGH* locus [19].

Screening is not without its technical challenges, not least because of the low frequency of pre-leukaemic cells. MI-FISH is very informative but technically demanding and time consuming. Ideally Q-PCR should be used, provided suitable genomic sequences are identified.

When the co-twin of a patient with leukaemia is clinically well, but has detectable, low level pre-leukaemic cells in the blood we have, in consultation with clinicians and parents, offered screening of the blood of the healthy co-twin every three months for one year (after the original diagnosis of ALL) and less frequently thereafter. The purpose of the screen is to determine if the level of putative pre-leukaemic cells remains very low or is increasing. Persistent low levels of loss of the putative pre-leukaemic cells is re-assuring for parents. If these cells do increase in frequency in blood, say by one or two orders of magnitude, then examination of bone marrow, the main site of clonal expansion, could be justified. The argument here is that early detection of an expanding leukemic clone provides an opportunity for the clinician to consider bone marrow examination and the option of early intervention. The premise however is that earlier treatment of a smaller sized leukaemic clone might be expected to be beneficial by decreasing the probability of drug resistance emerging. Formal evidence for this thesis is not available but we reported three cases of infant ALL in twins where early detection and treatment in the second twin was associated with a much better outcome than in the co-twin presenting with clinical disease [26].

Parents should be further advised that risk to the second twin is greatest in the first year to 18 months after the first twin was diagnosed and declines after that, screening of asymptomatic, healthy co-twins is probably unjustifiable after the age of 10, despite the very rare late occurrence of ALL in a co-twin of a patient with ALL, as in pair 7 above [8]. The evidence for suggesting this time frame is the observed difference in timing of diagnosis in a series of 19 cases of concordant ALL we recorded in children where all but one pair (# 7 here) were diagnosed within 18 months of each other [1].

The challenge for haematologists or oncologists discussing this issue with parents is compounded by the rarity of childhood leukaemia in twins and therefore a lack of experience plus the biological diversity of this group of cancers. Leukaemias other than B cell lineage ALL [21] can be pre-natal in origin and concordant twin cases with a common clonal origin have been reported for both T-ALL [32] and AML [20]. Twin pairs discordant for these leukaemia subtypes are also very likely to exist. The risk level for concordance in these other subtypes of childhood leukaemia is unknown but likely elevated, so testing of heathy co-twin blood would be prudent.

A succinct summary of the biology and risk level for leukaemia in twins written, in lay terms, for parents and their physicians is available at https://www.icr.ac.uk/leukaemia-in-twins.

## Data Availability

The datasets generated during and/or analysed during the current study are available from the corresponding author on reasonable request.
